# Effects of Vitamin A on Immune Responses and Vitamin A Metabolism in Broiler Chickens Challenged with Necrotic Enteritis

**DOI:** 10.3390/life13051122

**Published:** 2023-05-01

**Authors:** Shuangshuang Guo, Lai He, Yuanke Zhang, Junlong Niu, Changwu Li, Zhengfan Zhang, Peng Li, Binying Ding

**Affiliations:** Engineering Research Center of Feed Protein Resources on Agricultural By-Products, Ministry of Education, Hubei Key Laboratory of Animal Nutrition and Feed Science, Wuhan Polytechnic University, Wuhan 430023, China; guoshuang@whpu.edu.cn (S.G.); hlshasha@163.com (L.H.); zhangyk1@haid.com.cn (Y.Z.); lichangwu105@163.com (C.L.); 12523@whpu.edu.cn (Z.Z.); pengli@whpu.edu.cn (P.L.)

**Keywords:** vitamin A, retinoic acid, necrotic enteritis, immune response, metabolism, broiler chicken

## Abstract

**Simple Summary:**

Vitamin A is an anti-inflammatory vitamin and essential for the health of humans and animals. Necrotic enteritis is an enteric inflammatory disease in poultry caused by *Clostridium perfringens* infection. Due to the ban on using antibiotics in feed, necrotic enteritis has caused great economic losses in poultry production. Previous observations indicated that phospholipase C secreted by *C. perfringens* might interfere with the transformation of vitamin A into retinoic acid via the stimulation of prostaglandin E_2_ production, which impaired the modulation of vitamin A on immune responses. Therefore, the present study investigated the effects of dietary supplementation with a large dose of vitamin A on the immune responses and vitamin A metabolism in broilers suffering from necrotic enteritis and explored the underlying mechanisms. The results showed that vitamin A supplementation in chicken diets improved hepatic vitamin A deposition and modulated the expression of Th2 cell-related cytokines in the jejunum and spleen. In addition, dietary supplementation with vitamin A inhibited the expression of genes involved in the Janus kinase/signal transducer and activator of the transcription pathway and genes encoding retinoic acid receptors in the spleen of broilers. The present study indicated the modulatory effects of vitamin A supplementation in diets on the immune responses and vitamin A metabolism in necrotic enteritis-challenged broilers.

**Abstract:**

Necrotic enteritis (NE) is an important enteric inflammatory disease of poultry, and the effects of vitamin A (VitA) on NE birds are largely unknown. The present study was conducted to investigate the effects of VitA on the immune responses and VitA metabolism of NE broilers as well as the underlying mechanisms. Using a 2 × 2 factorial arrangement, 336 1-day-old Ross 308 broiler chicks were randomly assigned to 4 groups with 7 replicates. Broilers in the control (Ctrl) group were fed a basal diet without extra VitA supplementation. Broilers in the VitA group were fed a basal diet supplemented with 12,000 IU/kg of VitA. Birds in NE and VitA + NE groups were fed corresponding diets and, in addition, co-infected with *Eimeria* spp. and *Clostridium perfringens* on days 14 to 20. Samples of the blood, jejunum, spleen and liver were obtained on day 28 for analysis, and meanwhile, lesion scores were also recorded. The results showed that NE challenge increased lesion score in the jejunum and decreased serum glucose, total glyceride, calcium, phosphorus and uric acid levels (*p* < 0.05). VitA supplementation reduced the levels of serum phosphorus, uric acid and alkaline phosphatase in NE-challenged birds and increased serum low-density lipoprotein content and the activity of aspartate aminotransferase and creatine kinase (*p* < 0.05). Compared with the Ctrl group, the VitA and NE groups had higher mRNA expression of interferon-γ in the jejunum (*p* < 0.05). NE challenge up-regulated mRNA expression of interleukin (*IL*)-13, transforming growth factor-β4, aldehyde dehydrogenase (*RALDH*)-2 and *RALDH-3* in the jejunum, while VitA supplementation increased jejunal *IL-13* mRNA expression and hepatic VitA content, but down-regulated splenic *IL-13* mRNA expression (*p* < 0.05). The VitA + NE group had higher serum prostaglandin E_2_ levels and the Ctrl group had higher splenic *RALDH-3* mRNA expression than that of the other three groups (*p* < 0.05). NE challenge up-regulated jejunal retinoic acid receptor (*RAR*)-β and retinoid X receptor (*RXR*)-α as well as splenic *RAR-α* and *RAR-β* mRNA expression (*p* < 0.05). VitA supplementation up-regulated jejunal *RAR-β* expression but down-regulated mRNA expression of *RXR-α*, *RXR-γ*, signal transducers and activators of transcription (*STAT*) 5 and *STAT6* in the spleen (*p* < 0.05). Moreover, compared with the Ctrl group, the VitA and NE groups had down-regulated mRNA expression of jejunal and splenic Janus kinase (*JAK*) 1 (*p* < 0.05). In conclusion, NE challenge induced jejunal injury and expression of Th2 and Treg cell-related cytokines and enhanced *RALDH* and *RAR/RXR* mRNA expression, mainly in the jejunum of broilers. VitA supplementation did not alleviate jejunal injury or Th2 cell-related cytokine expression; however, it improved hepatic VitA deposition and inhibited the expression of *RALDH-3*, *RXR* and the JAK/STAT signaling pathway in the spleen of broilers. In short, the present study suggested the modulatory effects of vitamin A on the immune responses and vitamin A metabolism in broiler chickens challenged with necrotic enteritis.

## 1. Introduction

Vitamin A (VitA) is a micronutrient that is essential for maintaining vision, promoting growth and development, and protecting the integrity of intestinal mucosal epithelia. Furthermore, VitA is known as an anti-inflammation vitamin due to its critical role in enhancing immune function through the regulation of cellular and humoral immune processes [1]. VitA deficiency affects cytokine release and antibody production, reduces the production of natural killer cells, monocytes and macrophages, and impairs the maturation and proliferation of T- and B-lymphocytes [2]. It has been reported that broiler chickens fed VitA-deficient (400 IU/kg) diets developed a T helper (Th) 2 pro-inflammatory response, whereas chickens fed VitA-rich (15,000 IU/kg) diets exhibited a Th1 anti-inflammatory response [3]. 

The VitA is present as retinol, retinal and retinoic acid, among which retinoic acid shows the most biological activity. The conversion of retinol into retinal is a reversible step, catalyzed by the alcohol dehydrogenase (Adh) family. The aldehyde dehydrogenase (RALDH) family then catalyzes retinal to form retinoic acid, and this step is irreversible [4]. Retinoic acid is the ligand for the nuclear retinoic acid receptor (RAR), which acts as a ligand-activated transcription factor that heterodimerizes with retinoid X receptor (RXR) and regulates gene expression [5]. In mammals, retinoic acid supplementation increased *RALDH-2* expression and promoted the balance between Th17 and regulatory T (Treg) cell populations in the gut-associated lymphoid tissue in the presence of infection and inflammation [6,7]. Meanwhile, it was evidenced that retinoic acid exerted anti-inflammatory effects by inhibiting Janus kinase (JAK)/signal transducers and activators of the transcription (STAT) signaling pathway in rats [8,9]. 

Necrotic enteritis (NE) in chickens is an enteric disease due to *Clostridium perfringens* that has caused significant economic losses worldwide [10]. *C. perfringens* challenge triggers inflammatory responses in the broiler intestine by inducing Th2 and Th17 cytokine secretion [11], disrupting Th17/Treg homeostasis [12] and activating the JAK/STAT signaling pathway in the intestine of broiler chickens [13]. Both VitA and retinoic acid play critical roles in maintaining Th17/Treg balance in the intestine of mammals [14,15]. Coccidiosis is an important susceptibility factor to NE because the infection is known to increase mucogenesis and release essential amino acids from damaged tissue, thus providing nutrients for *C. perfringens* [16]. Adequate VitA (8000 IU/kg) has been reported to improve resistance to enteric diseases such as coccidiosis in broiler chickens [17,18]. Therefore, VitA might prevent NE by modulating immune responses in broilers.

It has been shown that type I phospholipase C secreted by *C. perfringens* can stimulate prostaglandin E_2_ (PGE_2_) production in organ-cultured rabbit gastric mucosa [19]. Furthermore, PGE_2_ can inhibit *RALDH* expression in mouse and human dendritic cells, and thus influence the immunomodulatory effects of VitA [20]. Therefore, it was speculated that VitA metabolism in NE birds might be interfered with by PGE_2_ production. In short, the aim of the present study was to investigate the effects of VitA at deficient (0 IU/kg) and high (12000 IU/kg) levels on the immune responses and VitA metabolism in NE birds and further explore the involvement of the JAK/STAT and RAR/RXR signaling pathways.

## 2. Materials and Methods

### 2.1. Experimental Design and Bird Management

All animal procedures used in the present study were approved by the Institutional Animal Care and Use Committee of Wuhan Polytechnic University (Number: WPU201904001). A 2 × 2 factorial randomized complete block design was used, and 336 1-day-old broiler chicks (Ross 308) were randomly assigned to 4 groups, each with 7 replicates of 12 birds (6 males and 6 females). The birds in the control (Ctrl) group were fed a corn–soybean meal basal diet including 1.05 mg/kg of carotene without extra VitA supplementation. According to the observation that a high level of VitA (15,000 IU/kg) showed great anti-inflammatory effects [3], birds in the VitA group were fed a basal diet supplemented with 12,000 IU/kg of VitA. Birds in NE and VitA + NE groups were fed corresponding diets and infected with *Eimeria* spp. and *C. perfringens*. The basal diet was formulated to meet or exceed the nutritional requirements of the National Research Council (1994) with the vitamin premix excluding VitA. The composition and nutrient levels of the basal diet are presented in Table 1. Retinol acetate, containing 500,000 IU/g of VitA, was used as the VitA source. Both the customized vitamin premix and retinol acetate were supplied by Blooming^®^ Biotechnology Co., Ltd. (Beijing, China). The analyzed dietary VitA levels were 11,970 IU/kg and 12,020 IU/kg in VitA and VitA + NE groups, respectively, which were determined using high-performance liquid chromatography (HPLC). The trial lasted for 28 days. Birds were raised in wire cages in an environmentally controlled room with 23 h light and were allowed ad libitum access to water and mashed diets throughout the trial.

### 2.2. Co-infection of Eimeria spp. and C. perfringens

A live attenuated trivalent coccidian vaccine (SCOCVAC^®^) used for white-feathered broilers, comprising 5 × 10^4^ oocysts of *Eimeria maxima* PMHY strain and *Eimeria tenella* PTMZ strain and 1 × 10^5^ oocysts of *Eimeria acervulina* PAHY strain, was obtained from Foshan Standard Bio-Tech Co., Ltd. (Foshan, China). Avian *C. pefringens* type A filed strain (CVCC2030) was obtained from China Veterinary Culture Collection Center (Beijing, China) and identified using tryptose–sulfite–cycloserine agar plates before use. The NE model was established as described by Wu et al. [21]. Briefly, at 14 days of age, birds in the challenged group were orally inoculated with a 20-fold dose of the coccidian vaccine. Unchallenged birds received an equal volume of sterile PBS. On days 18 to 20, each *Eimeria* spp.-inoculated bird was subsequently gavaged orally with 1 mL of actively growing culture of *C. perfringens* (3 × 10^8^ CFU/mL). Uninfected birds were given an equal volume of sterile medium. 

### 2.3. Sample Collection

Previous observations reported that higher jejunal lesion scores occurred at 7 days post-challenge of *Eimeria* spp. and *C. perfringens* than at 3 days post-challenge, and no significant difference in jejunal lesion scores was found between 7 and 17 days post-challenge [22]. Samples in the current study were collected on day 28. Two birds per replicate (fourteen birds per group) were randomly selected and blood was collected aseptically via the wing vein. The blood was centrifuged at 3000× *g*, 4 °C for 10 min to prepare serum, which was stored at −20 °C for the assay of biochemical and immune parameters as well as PGE_2_. Then, birds were euthanized by cervical dislocation. Approximately 1 cm of the mid-jejunum was sampled and immediately fixed in 4% paraformaldehyde for immunohistochemical analysis. The jejunum was longitudinally opened for NE lesion score. Jejunal mucosa and the spleen were sampled and stored at −80 °C for total RNA isolation. The liver was collected for the assay of VitA content.

### 2.4. Jejunal NE Lesion Score

The jejunal NE lesion score was performed on a scale from zero to six as described by Shojadoost et al. [10]. Briefly, 0 = no gross lesions; 1 = thin or friable walls, or diffuse superficial but removable fibrin; 2 = focal necrosis or ulceration with 1–5 foci, or non-removable fibrin deposit; 3 = focal necrosis or ulceration with 6–15 foci, or non-removable fibrin deposit; 4 = focal necrosis or ulceration with more than 16 foci, or non-removable fibrin deposit; 5 = variable patches of necrosis 2 to 3 cm long; 6 = diffuse and extensive necrosis typical of field cases. Two independent observers blinded to the experimental design performed the lesion scoring. 

### 2.5. Determination of Serum Biochemical Indices

Serum biochemical indices were measured using a Hitachi 7100 Automatic Biochemical Analyzer (Hitachi Instruments, Co., Ltd., Tokyo, Japan). The commercial kits obtained from Wako Pure Chemical Industries, Ltd. (Tokyo, Japan) were used for the assay of serum total protein, albumin, globulin, total bilirubin, glucose, total glyceride, total cholesterol, high-density lipoprotein, low-density lipoprotein, calcium, phosphorus, uric acid, aspartate aminotransferase, alanine aminotransferase, alkaline phosphatase, glutamyl transpeptidase and creatine kinase.

### 2.6. Serum Immunoglobulin (Ig) A and IgG Assay

Serum IgA and IgG measurements were conducted using commercially available ELISA kits (Bethyl Laboratories, Inc., Montgomery, TX, USA) according to the manufacturer’s protocol as Song et al. [12] described. The serum samples were appropriately diluted and added to anti-chicken IgA or IgG antibody pre-coated 96-well strip plates. After one hour of incubation at room temperature, the unbound proteins and molecules were washed off, and a biotinylated detection antibody was added to the wells for the binding of captured IgA or IgG. After incubation and washing, a streptavidin-conjugated horseradish peroxidase (HRP) was added. The plates were incubated for 30 min at room temperature and washed four times. Then, the TMB substrate solution was added to each well to develop a colorimetric reaction, which was stopped with the addition of dilute sulfuric acid. The absorbance of the yellow product at 450 nm is proportional to the amount of IgA or IgG analyte present in the samples and a four-parameter standard curve can be generated. The assay ranges for IgA and IgG measurements were 0.69–500 ng/mL and 1.37–1000 ng/mL, respectively. 

### 2.7. Immunohistochemical Analysis

The density of CD3^+^ intraepithelial T cells in the jejunal villus and crypt was analyzed with an indirect immunohistochemical method using a commercial kit obtained from Boster Biological Technology Co., Ltd., Wuhan, China [23]. After fixing, sectioning and blocking, the jejunal samples were incubated overnight at 4 °C with rat-anti human CD3 antibody (1:100 dilution), which cross-reacts with chicken CD3 complex. Then, the jejunal sections were incubated with a secondary antibody conjugated with HRP and the CD3^+^ T cells were visualized using chromogenic dye. Finally, the CD3^+^ T cells were quantified using a light microscope (Olympus, Tokyo, Japan), which was equipped with a digital camera (Olympus, Tokyo, Japan) and an image analysis program (ProRes CapturePro software, Jenoptik, Jena, Germany). Data were presented as the number of CD3^+^ T cells per 1000 μm^2^ of villus or crypt regions. 

### 2.8. Measurement of VitA Content in Liver

The concentration of VitA in the liver was analyzed according to the procedure described by Idi et al. [24] with modifications. Approximately 3 g of the liver samples were homogenized on an ice bath with 6 mL of 96% ethanol (*v*/*v*). The homogenate was further saponified with methanol, 20% ascorbic acid (*w*/*v*) and potassium hydroxide solution (1:1, *w*/*v*). Finally, retinol was extracted with petroleum ether and diethyl ether (1:1, *v*/*v*), and the extract was subsequently injected into the HPLC system. 

The Waters Breeze HPLC system (Waters Corporation, Milford, MA, USA), which comprises 1525 binary HPLC pumps, a 2487 Dual-λ absorbance detector, a 717 plus autosampler, Breeze system software and a chromatographic column (Waters XBridge C18, 3.5 μm, 4.6 mm × 250 mm), was used. Identification and quantification of retinol were obtained using a comparison of retention time as well as peak areas with external standards. The mobile phase consisted of water and methanol with a flow rate of 0.8 mL/min. An ultraviolet detector was performed with a wavelength of 325 nm. The content of VitA was expressed as milligrams of retinol per 100 g of wet tissue. 

### 2.9. Serum PGE_2_ Assay

The serum PGE_2_ level was measured using a commercial ELISA kit obtained from R&D Systems, Inc. (Minneapolis, MN, USA). The assay was conducted according to the instructions of the manufacturer. Briefly, in the first incubation at room temperature for 2 h, PGE_2_ in serum bound to the antibody coated in a 96-well plate. During the second incubation at room temperature for 30 min, HRP-labeled PGE_2_ bound to the remaining antibody sites. After washing to remove the unbound HRP-labeled PGE_2_, a substrate solution was added to each well for the determination of bound HRP activity. The color development was stopped, and the absorbance was read at 450 nm. The intensity of color was inversely proportional to the concentration of PGE_2_ in serum.

### 2.10. RNA Isolation and Quantitative Real-Time PCR

Total RNA was isolated from the jejunum, spleen and liver samples using Trizol reagent (Invitrogen Life Technologies, Carlsbad, CA, USA) as previously described [23]. The concentration and purity of total RNA were checked using a NanoDrop^®^ ND-2000 UV-VIS spectrophotometer (Thermo Scientific, Wilmington, DE, USA). The RNA integrity was verified using agarose gel electrophoresis. One microgram of total RNA was reverse transcribed using the PrimeScript® RT reagent Kit with gDNA Eraser (Takara Biotechnology (Dalian) Co., Ltd., Dalian, China). The quantitative real-time PCR was conducted with a 7500-fluorescence detection system (Applied Biosystems, Foster City, CA, USA) using the SYBR Premix Ex TaqTM kit (Takara Biotechnology (Dalian) Co., Ltd.). The primer pairs for the amplification of cytokine genes (*INF-γ*, *IL-13*, *IL-17* and *TGF-β4*), retinoic acid synthesis-associated genes (*Adh-1*, *RALDH-2* and *RALDH-3*), key genes in the JAK/STAT signaling pathway (*JAK1*, *JAK2*, *STAT1*, *STAT5* and *STAT6*) and retinoic acid receptor genes (*RAR-α*, *RAR-β*, *RAR-γ*, *RXR-α* and *RXR-γ*) in the jejunum and spleen are presented in Table 2. The β-actin served as a housekeeping gene. The PCR conditions were an initial denaturation step at 95 °C for 30 s then 40 cycles at 95 °C for 5 s, and the annealing and extension temperatures ranged between 58 and 62 °C for 34 s (Table 2). Each biological sample was run in triplicate. Gene expression was quantified using the comparative threshold cycle method, and the data were expressed as relative values to the Ctrl group [25]. 

### 2.11. Statistical Analysis

The data were analyzed with a two-factorial ANOVA using a univariate general linear model with SPSS version 21.0 (SPSS Inc., Chicago, IL, USA). Differences among individual treatment means were tested using Duncan’s multiple comparison when significant interactions between VitA supplementation and NE challenge were observed. Pearson correlation analysis of significant data from the serum, jejunal and hepatic samples was conducted and shown in a heat map. Results are presented as the mean ± SEM. *p* < 0.05 was considered significant.

## 3. Results

### 3.1. Jejunal NE Lesion Score

**Figure 1:**
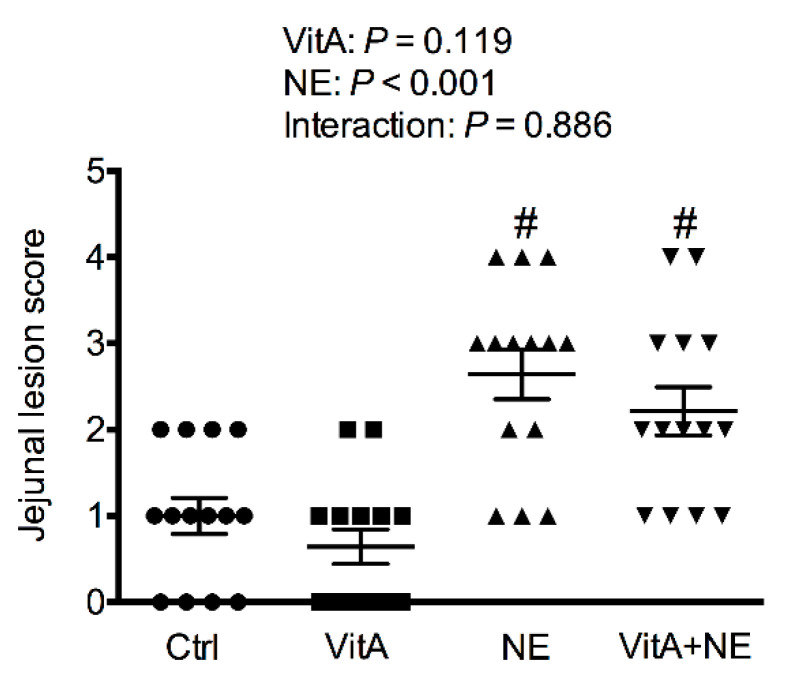
The NE lesion score for the jejunum. Circles, squares, forward triangles and inverted triangles indicate individual samples in Ctrl, VitA, NE and VitA + NE groups, respectively. Ctrl, control; VitA, vitamin A; NE, necrotic enteritis. ^#^ Indicates the significant main effects of NE challenge vs. unchallenged (*p* < 0.05).

As shown in Figure 1, co-infection with *Eimeria* spp. and *C. perfringens* significantly increased the jejunal NE lesion score both in the NE and VitA + NE groups compared to the non-infected groups (*p* < 0.05). In addition, dietary supplementation with VitA did not affect the jejunal lesion score. 

### 3.2. Serum Biochemical Indices

As presented in Table 3, dietary supplementation with VitA significantly increased serum low-density lipoprotein levels and the activity of aspartate aminotransferase and creatine kinase, and it also reduced serum phosphorus and uric acid contents (*p* < 0.05). NE challenge significantly decreased the levels of serum glucose, total glycerides, calcium, phosphorus and uric acid (*p* < 0.05). An interaction was found in serum alkaline phosphatase activity between VitA supplementation and NE challenge (*p* < 0.05). VitA supplementation decreased serum alkaline phosphatase activity in NE-challenged broilers (*p* < 0.05), while it had no effect on the unchallenged birds (*p* > 0.05).

### 3.3. Serum Immune Parameters 

**Figure 2:**
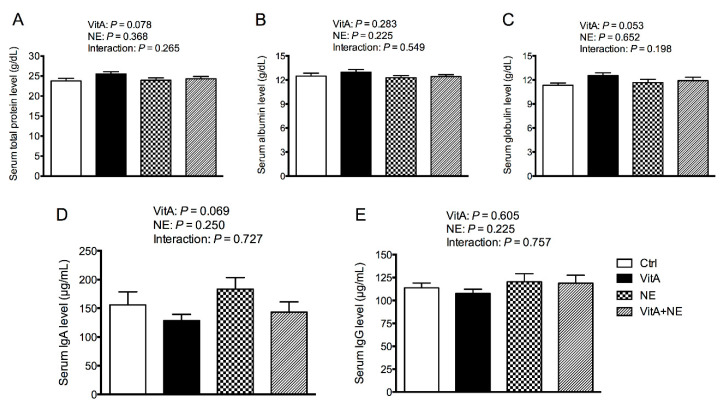
Serum immune parameters in broiler chickens among groups. Serum total protein (**A**), albumin (**B**), globulin (**C**), IgA (**D**) and IgG (**E**) levels were analyzed. Data are expressed as mean and SE from 14 chickens. Ig, immunoglobulin; Ctrl, control; VitA, vitamin A; NE, necrotic enteritis.

As shown in Figure 2, serum total protein, albumin, globulin, IgA and IgG contents were not significantly affected by VitA supplementation or NE challenge (*p* > 0.05). In addition, VitA supplementation tended to increase serum total protein (*p* = 0.078) and globulin (*p* = 0.053) levels. 

### 3.4. Jejunal CD3^+^ T Cell Density

**Figure 3:**
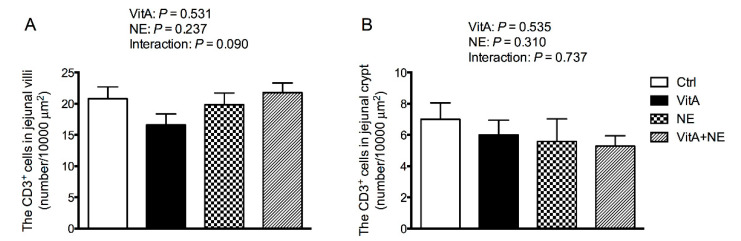
The CD3^+^ T cell counts in jejunal villus (**A**) and crypt (**B**) in broiler chickens among groups. Data are expressed as mean and SE from 14 chickens. Ctrl, control; VitA, vitamin A; NE, necrotic enteritis.

The CD3^+^ T cell density in the jejunal villi and crypts among groups was not significantly affected either by VitA supplementation or NE challenge, as show in Figure 3 (*p* > 0.05).

### 3.5. Immune Gene Expression in the Jejunum and Spleen

**Figure 4:**
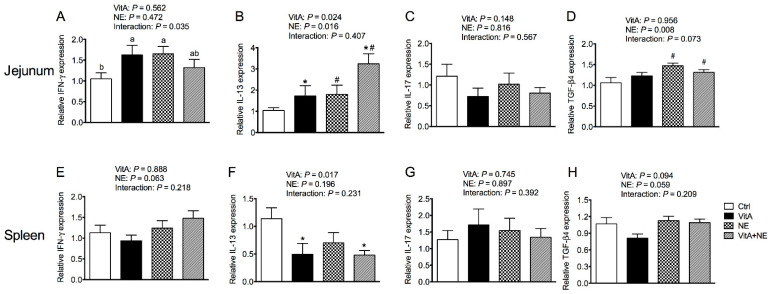
The mRNA expression of cytokines in the jejunum (**A**–**D**) and spleen (**E**–**H**) in broiler chickens among groups. Data are expressed as mean and SE from 14 chickens. ^a,b^ Bars with different letters differ significantly (*p* < 0.05). * Indicates the significant main effects of VitA supplementation vs. no supplementation (*p* < 0.05). ^#^ Indicates the significant main effects of NE challenge vs. unchallenged (*p* < 0.05). *IFN-γ*, interferon-γ; *IL*, interleukin; *TGF-β4*, transforming growth factor-β4; Ctrl, control; VitA, vitamin A; NE, necrotic enteritis.

VitA supplementation increased the relative mRNA expression of *IL-13* in the jejunum (*p* < 0.05) (Figure 4B). NE challenge increased both the relative mRNA expression of *IL-13* and *TGF-β4* in the jejunum (*p* < 0.05) (Figure 4B,D). In addition, an interaction was found in the relative mRNA expression of *IFN-γ* in the jejunum among groups (*p* < 0.05). VitA supplementation increased jejunal *IFN-γ* mRNA expression in unchallenged birds (*p* < 0.05) but did not significantly affect it in NE-challenged birds (*p* > 0.05) (Figure 4A). Regarding the spleen, the VitA-supplemented diet down-regulated the mRNA expression of *IL-13* (*p* < 0.05) (Figure 4F). NE challenge tended to increase the mRNA expression of *IL-13* (*p* = 0.063) and *TGF-β4* (*p* = 0.059) (Figure 4E,H). Moreover, Jejunal IL-17 as well as splenic IFN-γ, IL-17 and *TGF-β4* expression was not significantly influenced by the treatments (*p* > 0.05) (Figure 4C,E,G,H).

### 3.6. Gene Expression of the JAK-STAT Signaling Pathway Components in the Jejunum and Spleen

**Figure 5:**
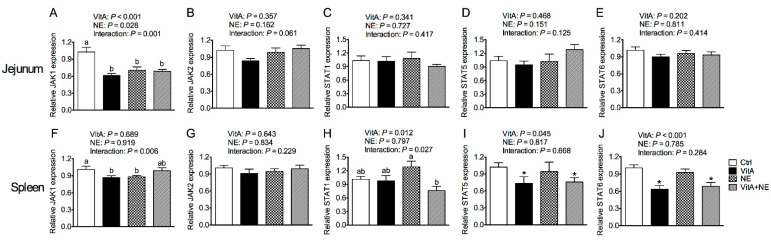
The mRNA expression of genes involved in the JAK/STAT signaling pathway in the jejunum (**A**–**E**) and spleen (**F**–**J**) in broiler chickens among groups. Data are expressed as mean and SE from 14 chickens. ^a,b^ Bars with different letters differ significantly (*p* < 0.05). * Indicates the significant main effects of VitA supplementation vs. no supplementation (*p* < 0.05). *JAK*, Janus kinase; *STAT*, signal transducer and activator of transcription; Ctrl, control; VitA, vitamin A; NE, necrotic enteritis.

VitA supplementation and NE challenge showed interactive effects on the mRNA expression of *JAK1* in the jejunum (*p* < 0.05) (Figure 5A). VitA supplementation down-regulated jejunal *JAK1* mRNA expression in unchallenged birds (*p* < 0.05) and did not significantly affect it in challenged birds (*p* > 0.05). The gene expression of *JAK2* and *STAT*s in the jejunum was not significantly affected by the treatments (*p* > 0.05) (Figure 5B–E). VitA supplementation and NE challenge had interactive effects on *JAK1* and *STAT1* mRNA expression in the spleen (*p* < 0.05) (Figure 5F,H). VitA supplementation down-regulated splenic *JAK1* and *STAT1* mRNA expression in unchallenged and challenged birds, respectively (*p* < 0.05). Moreover, VitA supplementation down-regulated *STAT5* and *STAT6* mRNA expression in the spleen (*p* < 0.05) (Figure 5I,J). The gene expression of splenic *JAK2* was not significantly influenced (*p* > 0.05) (Figure 5G).

### 3.7. Hepatic VitA Content and Serum PGE_2_ Level

**Figure 6:**
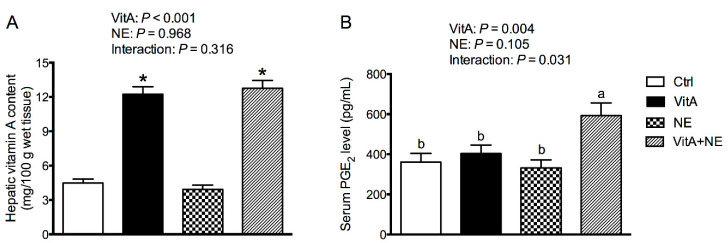
The hepatic VitA content (**A**) and serum PGE_2_ level (**B**) in broiler chickens among groups. Data are expressed as mean and SE from 14 chickens. ^a,b^ Bars with different letters differ significantly (*p* < 0.05). * Indicates the significant main effects of VitA supplementation vs. no supplementation (*p* < 0.05). Ctrl, control; VitA, vitamin A; NE, necrotic enteritis; PGE_2_, prostaglandin E_2_.

Significantly higher concentrations of VitA in the liver were identified in both the VitA and VitA + NE groups than those in birds in Ctrl and NE groups (*p* < 0.001) (Figure 6A). Additionally, VitA supplementation and NE challenge had an interactive effect on serum PGE_2_ level, and as shown in Figure 6B, VitA supplementation increased serum PGE_2_ content in NE-challenged birds (*p* < 0.05) but did not significantly affect it in unchallenged birds (*p* > 0.05).

### 3.8. The Relative mRNA Expression of Genes Involved in Retinoic Acid Synthesis in the Jejunum and Spleen 

**Figure 7:**
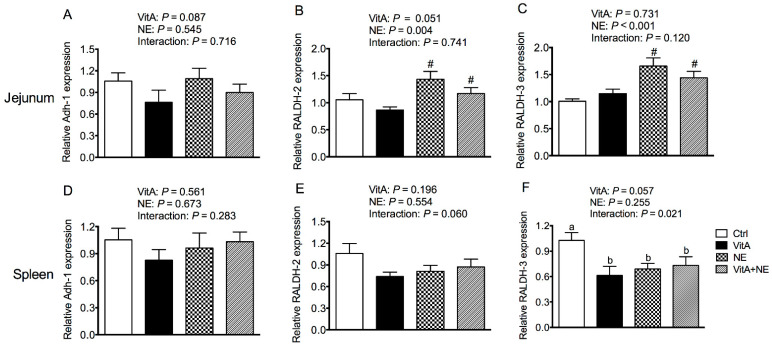
The mRNA expression of genes involved in retinoic acid synthesis in the jejunum (**A**–**C**) and spleen (**D**–**F**) in broiler chickens among groups. Data were expressed as mean and SE from 14 chickens. ^a,b^ Bars with different letters differ significantly (*p* < 0.05). ^#^ Indicates the significant main effects of NE challenge vs. unchallenged (*p* < 0.05). *Adh-1*, aldehyde dehydrogenase-1; *RALDH*, retinal dehydrogenase; Ctrl, control; VitA, vitamin A; NE, necrotic enteritis.

Figure 7B,C show that NE challenge significantly up-regulated *RALDH-2* and *RALDH-3* relative mRNA expression in the jejunum (*p* < 0.05). VitA supplementation and NE challenge showed an interactive effect on *RALDH-3* mRNA expression in the spleen (*p* < 0.05) (Figure 7F). In detail, VitA supplementation down-regulated *RALDH-3* mRNA expression in the spleen of unchallenged broilers (*p* < 0.05) but did not significantly influence it in their challenged counterparts (*p* > 0.05). In addition, the relative *Adh-1* mRNA expression in the jejunum and spleen as well as *RALDH-2* expression in the spleen was not significantly affected by the treatments (*p* > 0.05) (Figure 7A,D,E). 

### 3.9. The Relative mRNA expression of Genes Encoding Retinoic Acid Receptors in the Jejunum and Spleen 

**Figure 8:**
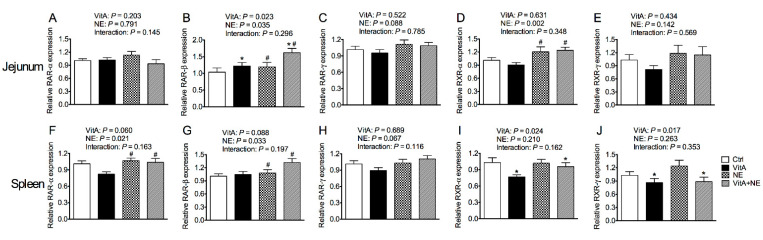
The mRNA expression of genes encoding *RAR* and *RXR* in the jejunum (**A**–**E**) and spleen (**F**–**J**) in broiler chickens among groups. Data are expressed as mean and SE from 14 chickens. *Indicates the significant main effects of VitA supplementation vs. no supplementation (*p* < 0.05). ^#^Indicates the significant main effects of NE challenge vs. unchallenged (*p* < 0.05). *RAR*, retinoic acid receptor; *RXR*, retinoid receptor X; Ctrl, control; VitA, vitamin A; NE, necrotic enteritis.

VitA supplementation increased the mRNA expression of *RAR-β* in the jejunum of broiler chickens (*p* < 0.05) (Figure 8B). NE challenge up-regulated the mRNA expression of *RAR-β* and *RXR-α* in the jejunum of broiler chickens (*p* < 0.05) (Figure 8B,D). On the other hand, VitA supplementation down-regulated the mRNA expression of *RXR-α* and *RXR-γ* in the spleen (*p* < 0.05) (Figure 8I, J). In contrast, NE challenge increased splenic *RAR-α* and *RAR-β* mRNA expression (*p* < 0.05) (Figure 8F,G). Although, the relative mRNA expression of *RAR-α, RAR-γ* and *RXR-γ* in the jejunum as well as *RAR-γ* in the spleen was not significantly influenced (*p* > 0.05) (Figure 8A,C,E,H). 

### 3.10. Pearson Correlation Analysis of Parameters

**Figure 9:**
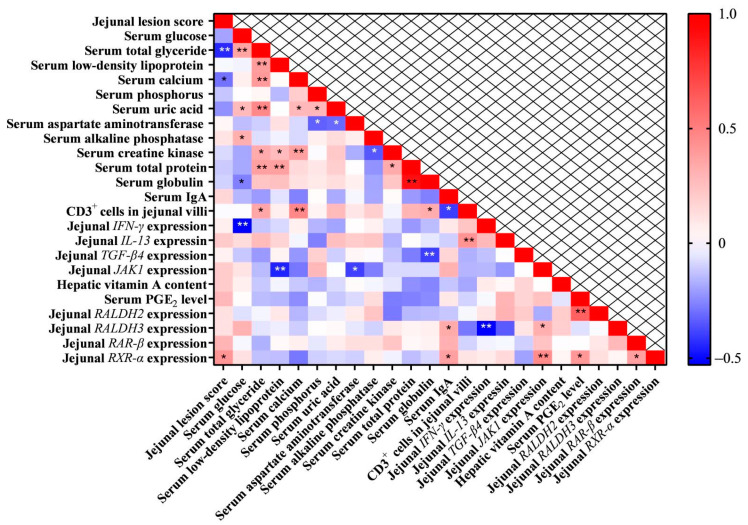
The heat map showing the Pearson correlation analysis among parameters. Pearson correlation coefficients are indicated with the colored bar from blue to red. The significance of Pearson correlation coefficients is indicated with asterisks, where * *p* < 0.05 and ** *p* < 0.01.

As shown in Figure 9, there was a significant positive correlation among most serum biochemical indices, but they exhibited a negative correlation with jejunal NE lesion score as well as cytokine and *JAK1* expression. CD3^+^ cell counts in the jejunal villi were positively correlated with serum biochemical indices but negatively correlated with serum IgA content. Jejunal *RALDH* and *RAR/RXR* expression showed a positive correlation with serum IgA and PGE_2_ levels and *JAK1* expression. 

## 4. Discussion

NE is a multifactorial disease commonly seen in 2–5-week-old broiler chickens. The clinical form of NE is characterized by diarrhea and high mortality, and subclinical NE is usually unnoticed with poor growth performance due to subtle epithelial damage leading to impaired nutrient absorption [26]. The jejunal lesion score increased with NE challenge in the present study, indicating the successful establishment of NE. This was consistent with the decrease in feed intake and impairment of the intestinal barrier function in NE-challenged birds [27]. At the same time, serum glucose, total glyceride, calcium, phosphorus and uric acid levels were reduced with NE challenge as well. This suggested that the metabolism of carbohydrates, lipids, proteins and minerals might be interfered with by NE challenge. Xue et al. [28] reported that in a NE model for broilers induced with *Eimeria* spp. and *C. perfringens* co-infection, the serum cholesterol and uric acid contents were reduced in challenged birds. In the present study, the decrease in phosphorus and uric acid levels and increase in aspartate aminotransferase and creatine kinase activities induced with VitA supplementation indicated that excess VitA in the diet caused hepatic, cardiac and renal damage [29]. On the contrary, VitA supplementation increased serum low-density lipoprotein content regardless of NE challenge and reduced alkaline phosphatase activity in NE-challenged birds, which exhibited beneficial effects on lipid transportation and hepatobiliary function. Considering the crucial role of the liver in lipid metabolism, more studies should explore how VitA influences liver function in NE-challenged broiler chickens.

VitA plays an essential role in the regulation of innate and cell-mediated immunity [2]. In the present study, VitA supplementation tended to increase serum total protein and globulin levels but decrease IgA content. This suggested that VitA tended to enhance non-specific immune response without IgA involvement. VitA has been demonstrated to play a critical role in immunoglobulin synthesis, and dietary deficiency of VitA is associated with impaired IgA and IgG synthesis [30]. This phenomenon was not observed in the present study. Similarly, Rombout et al. [30] reported that plasma IgA was not significantly affected by dietary VitA deficiency or supplementation. It was reported that local cell-mediated immunity could be modulated by VitA as well. Dalloul et al. [17] observed that VitA deficiency decreased the CD3^+^ T cell population in the small intestine of broilers with and without *E. acervulina* challenge. CD3^+^ T cells within the intestinal epithelium are an important component of the local immune system, and the increase in CD3^+^ T cells indicates a strong resistance to enteric diseases [23]. Alizadeh et al. [31] reported that in ovo administration of retinoic acid increased the percentage of CD3^+^CD8^+^ T cells in the spleen of neonatal chickens at 5 and 10 days post-hatch. In the current study, CD3^+^ T cell density in the jejunal villi and crypts was not significantly influenced by VitA treatment. This might be attributed to the mature immune system of broilers at 28 days of age and the low severity of NE. In addition, CD3^+^ T cell density may also be influenced by different tissue sources.

In NE chickens, the innate immune responses mediated by TLR signaling activation lead to the maturation of dendritic cells and the subsequent differentiation of naïve T helper cells into mature effector cells with diverse functions, such as Th1, Th2, Th17 and Treg, which secrete IFN-γ, IL-13, IL-17 and TGF-β, respectively [32]. In the present study, NE challenge significantly up-regulated the jejunal *IL-13* and *TGF-β4* mRNA expression, which suggested that Th2 and Treg cells might be involved in the adaptive immunity against *Eimeria* spp. and *C. perfringens* infection. Similarly, the previous observation showed that *C. perfringens* challenge significantly increased the mRNA expression of *IL-1β* and *TGF-β4* in the jejunum of broiler chickens [23]. Sersun Calefi et al. [33] reported that *Eimeria* spp. and *C. perfringens* co-infection was highly correlated with *IFN-α*, *IFN-γ*, *IL-4*, *IL-13*, *IL-16*, *LITAF*, *TGF-β4* and *TNFSF15* in the jejunum, indicating that Th2 type cytokines mainly participated in avian NE. The current study showed that VitA supplementation up-regulated jejunal *IFN-γ* expression in unchallenged birds. Consistently, Dalloul et al. [17] reported that VitA deficiency decreased serum *IFN-γ* levels and local immune defenses, leading to low resistance to *E. acervulina* infection. The present study observed that VitA exhibited opposite effects on *IL-13* mRNA expression in the jejunum and spleen. VitA supplementation up-regulated jejunal *IL-13*, while it down-regulated splenic *IL-13*. Consistently, Shojadoost et al. [34] reported that in ovo inoculation of 270 μmol/egg of retinoic acid down-regulated the relative expression of *IL-13* in the spleen of chicken embryos at 24 h post-inoculation. In contrast, Fan et al. [35] demonstrated that dietary supplementation with 12,000 IU/kg VitA up-regulated *IL-13* mRNA expression in the lungs of broilers. This suggested that VitA showed tissue-specific effects on *IL-13* expression, indicating its enhancement and suppression of Th2-related cytokine expression in the jejunum and spleen of broilers, respectively. The pathogen infection stimulated pro-inflammatory responses with *IL-13* expression. Lessard et al. [3] demonstrated that chickens fed a highly VitA-enriched diet (15000 IU/kg) developed a Th1 rather than a Th2 immune response mainly in the spleen. 

The JAK/STAT signaling pathway is implicated in the pathogenesis of inflammatory and autoimmune diseases, and it can be activated by many cytokines [36]. In the classical singling pathway, cytokine binding of its cognate receptor leads to receptor dimerization followed by docking of JAK and consequent hetero- and homodimerization of STAT. Activated STAT then undergoes translocation to the nucleus to bind DNA elements to regulate the transcription of associated genes [37]. It has been reported that *E. maxima* and *C. perfringens* co-infection activate the JAK/STAT signaling pathway in the ileal and splenic innate immune response of NE broilers, especially the Marek’s disease-susceptible chicken line 7.2 [38,39]. In the present study, NE challenge did not significantly up-regulate the expression of *JAK*s and *STAT*s, which might be due to the low severity of NE. Interestingly, on the contrary, it was observed that a VitA-supplemented diet down-regulated jejunal and splenic *JAK1* in unchallenged broilers, splenic *STAT1* in challenged birds and splenic *STAT5* and *STAT6* regardless of the challenge. Choi et al. [9] reported that 9-cis-retinoic acid and all-trans-retinoic acid increased the mRNA expression of suppressors of cytokine signaling (SOCS), which are negative regulators of the JAK/STAT pathway, and thereby inhibited IFN-γ-induced activation of JAK1, JAK2, STAT1 and STAT3 in astrocytes. Uniyal et al. [8] observed that all-trans retinoic acid ameliorated inflammation and improved alveolar epithelium regeneration by interfering with the normal binding of ligands and receptors in the ERK and JAK/STAT pathways in the lungs of emphysematous rats. The suppression of JAK/STAT in the current study might be attributed to retinoic acid or VitA metabolites, but the underlying mechanism needs further investigation. Combining the results from immune responses in serum, jejunum, and spleen, the present study indicated the modulatory effects of vitamin A supplementation in diets on the immune responses in NE-challenged broilers.

Given the significance of the liver on metabolism in the body, it is, therefore, necessary to continue to investigate the deposition status of VitA in the liver. The liver is the major storage site for VitA, containing 80% of the total body reserves when VitA status is normal [40]. It was as expected that birds fed the VitA-supplemented diet had much higher hepatic VitA content in the current study. Yuan et al. [41] reported that supplementation with increasing levels of VitA linearly increased VitA in the liver and yolk of broiler breeders. Retinol and retinyl ester are dietary forms of VitA but not biologically active. They need transformation by Adh and RALDH to become retinoic acid [42]. The intestine is another important part regulating the VitA metabolism. The present study showed that NE challenge up-regulated *RALDH-2* and *RALDH-3* in the jejunum. This was contradictory to previous speculation that *C. perfringens* challenge might suppress *RALDH* expression via PGE_2_ production. The serum PGE_2_ level was elevated in the challenged birds fed the VitA-supplemented diet in the present study. Cattin et al. [43] reported that bacterial/fungal pathogens in the gut promoted RALDH activity in monocyte-derived dendritic cells, which boosted HIV infection and outgrowth in CD4^+^ T cells. Broadhurst et al. [44] demonstrated that *Schistosoma mansoni* infection up-regulated *RALDH2* to induce retinoic acid synthesis in alternatively activated macrophages during retinoic acid-dependent Th2 immunity in mice. Therefore, the up-regulation of jejunal *RALDH* in NE-challenged broilers might be a way to produce retinoic acid for enhancing immune responses against pathogens and parasites. The content of retinoic acid should be determined in further investigations. The present study revealed that VitA supplementation down-regulated splenic *RALDH-3* expression in unchallenged birds and showed no significant effects on the decrease in splenic *RALDH-3* expression in NE birds. It was evidenced that supplementation with a mixture of VitA and 10% all-trans-retinoic acid exhibited excess retinol availability in the lung of rats, resulting in decreased uptake of VitA and production of retinoic acid through the down-regulated expression of *RALDH* [45]. The relationship between nutrition and the liver–spleen axis has recently been emphasized, which includes the role of dietary supplementation with vitamins [46]. The conversion of VitA to retinoic acid in the spleen was scarcely reported.

Retinoic acid is the main active form of VitA, and it is transported to the nucleus after synthesis and recognized by RAR/RXR heterodimers bound to retinoic acid response elements where they regulate the transcriptional progression of various genes [47]. The present study observed that the VitA-supplemented diet up-regulated jejunal *RAR-β* and down-regulated splenic *RXR-α* and *RXR-γ*, indicating a tissue-specific response. Krüger et al. [48] demonstrated that feeding calves a milk-based formula with VitA did not significantly affect jejunal *RAR* and *RXR* mRNA levels, but it increased hepatic *RAR-β* expression compared to counterpart diets without VitA. It was demonstrated that VitA deficiency down-regulated *RAR-α* mRNA in the intestinal mucosa of rats and thereby failed to modulate mucosal immunity [49]. Few studies in the literature reported the effects of VitA or retinoic acid on *RAR* and *RXR* expression in the spleen. In the current study, NE challenge up-regulated jejunal *RAR-β* and *RXR-α* as well as splenic *RAR-α* and *RAR-β*. This was consistent with the up-regulation of *RALDH* expression with NE challenge. Luo et al. [50] evidenced that the expression of the nuclear receptors *RXR-α* and peroxisome proliferator-activated receptor α was decreased in the inflamed colonic mucosa of ulcerative colitis patients and in IL-1β-treated Caco2-BBE cells. Further investigation on *RAR* and *RXR* expression in NE birds is needed in the future.

The Pearson correlation analysis of serum and jejunal parameters greatly reflected the effects of NE challenge on these parameters. As NE challenge decreased serum biochemical indices and increased the jejunal lesion score as well as cytokine expression, there was a positive correlation among most serum biochemical indices, which were negatively correlated with jejunal lesion score and cytokine expression. NE challenge up-regulated jejunal *RALDH* and *RAR/RXR* expression, which showed a positive correlation with serum PGE_2_ levels. Meanwhile, jejunal villus CD3^+^ cell counts positively correlated with serum biochemical indices and negatively correlated with serum IgA content. This suggested that local and systematic immune responses to NE challenge differed. Jejunal JAK1 expression negatively correlated with serum biochemical indices and positively correlated with jejunal *RALDH3* and *RXR-α* expression. This indicated the involvement of the JAK/STAT and RAR/RXR signaling pathways in NE broilers fed the VitA-supplemented diet. 

## 5. Conclusions

NE challenge induced jejunal injury, Th2 and Treg cell-related cytokine expression and enhanced *RALDH* and *RAR/RXR* expression mainly in the jejunum of broilers. VitA supplementation improved hepatic VitA deposition and did not alleviate jejunal injury and Th2 cell-related cytokine expression, but it inhibited the expression of *RALDH-3*, *RXR* and the JAK/STAT signaling pathway in the spleen of broilers. The present study indicated the modulatory effects of VitA supplementation in diets on the immune responses and VitA metabolism in NE-challenged broilers. 

## Figures and Tables

**Table 1:** The composition and nutrient levels of basal diets.

Item (%, Unless Otherwise Indicated)	Starter Diet (d 1–21)	Grower Diet (d 22–28)
Ingredients		
Corn (crude protein, 7.8%)	51.73	57.68
Soybean meal (crude protein, 43.0%)	40.73	35.15
Soybean oil	3.36	3.66
Dicalcium phosphate	1.92	1.33
Limestone	1.16	1.26
Sodium chloride	0.35	0.35
DL-Methionine (98%)	0.26	0.13
Choline chloride (50%)	0.25	0.20
Vitamin premix ^1^	0.04	0.04
Trance mineral premix ^2^	0.20	0.20
Calculated nutrient levels		
Metabolic energy (Mcal/kg)	2.92	3.00
Crude protein	21.50	19.50
Calcium	1.00	0.90
Available Phosphorus	0.45	0.35
Lysine	1.17	1.04
Methionine	0.57	0.40
Threonine	0.82	0.74

^1^ The vitamin premix supplied the following per kilogram of diet: vitamin D_3_, 2500 IU; vitamin K_3_ 2.65 mg; vitamin E, 30 IU; vitamin B_1_, 2 mg; vitamin B_2_, 6 mg; vitamin B_12_, 0.025 mg; biotin, 0.0325 mg; folic acid, 1.25 mg; pantothenic acid, 12 mg; nicotinic acid, 50 mg. ^2^ The trace mineral premix supplied the following per kilogram of diet: copper, 8 mg; iron, 80 mg; zinc, 75 mg; manganese, 100 mg; selenium, 0.15 mg; iodine, 0.35 mg.

**Table 2:** Primers used for quantitative real-time PCR.

Gene Name	Accession Number	Forward Sequence (5′-3′)	Reverse Sequence (5′-3′)	Annealing and Extension Temperature (°C)
*β-actin*	NM_205518	GAGAAATTGTGCGTGACATCA	CCTGAACCTCTCATTGCCA	60
*IFN-γ*	Y07922	AGCTGACGGTGGACCTATTATT	GGCTTTGCGCTGGATTC	60
*IL-13*	AJ621735	CCAGGGCATCCAGAAGC	CAGTGCCGGCAAGAAGTT	62
*IL-17*	AJ493595	CTCCGATCCCTTATTCTCCTC	AAGCGGTTGTGGTCCTCAT	62
*TGF-β4*	M31160	CGGGACGGATGAGAAGAAC	CGGCCCACGTAGTAAATGAT	60
*JAK1*	XM_015290965	TGCACCGTGACTTAGCAGCAAG	TCTGAATCAAGCATTCTGGAGCATACC	60
*JAK2*	XM_015280061	TCGCTATGGCATTATTCG	GTGGGGTTTGGTCCTTTT	60
*STAT1*	XM_015289392	TAAAGAGGGAGCAATCAC	ATCAGGGAAAGTAACAGC	60
*STAT5*	XM_015299590	TCCCACCTGGAGGATTCA	TTCTTCAGCACCTCCATCAC	60
*STAT6*	XM_015274736	GCAACCTCTACCCCAACA	TCCCTTTCGCTTTCCACT	62
*Adh-1*	NM_204577	GAAGGAGCTGGGATTGTG	GCTGCATTCTCCACACTG	58
*RALDH-2*	AF064253	CAAGACATGAACCCATCG	GAGCTGGAGCAATCTTCC	60
*RALDH-3*	AF246710	AGGCAGCATTCCAGAGAG	TCAGCCAGCTTGTGTAGG	60
*RAR-α*	NM_204536	AGGAGCTGATCGAGAAGG	GAGCTGTTGTTCGTGGTG	60
*RAR-β*	NM_205326	GCATCAGTGCAAAAGGTG	TGTCAGTGGTTCGTGTCC	60
*RAR-γ*	NM_205294	GATGAAGATCACCGACCTG	TCCTCCTCGAACATCTCG	60
*RXR-α*	NM_204536	GATGCGAGACATGCAGATG	GTCGGGGTATTTGTGCTTG	60
*RXR-γ*	NM_205294	CCAAGACGGAGGC ATACAG	GGAGCGATGGGAGAAGGAT	60

*IFN-γ*, interferon-γ; *IL*, interleukin; *TGF-β4*, transforming growth factor-β4; *Adh-1*, aldehyde dehydrogenase-1; *RALDH*, retinal dehydrogenase; *RAR*, retinoic acid receptor; *RXR*, retinoid receptor X; *JAK*, Janus kinase; *STAT*, signal transducer and activator of transcription.

**Table 3:** Effects of VitA on serum biochemical indices for NE-challenged broilers.

Item	Ctrl	VitA	NE	VitA + NE	SEM	*p*-Values		
						VitA	NE	VitA × NE
Total bilirubin (mg/dL)	1.14	1.03	1.07	1.06	0.02	0.145	0.665	0.263
Glucose (mg/dL)	255.23	253.46	245.45	236.21	2.31	0.204	0.003	0.386
Total glyceride (mg/dL)	44.54	55.71	36.92	32.70	2.53	0.453	0.002	0.100
Total cholesterol (mg/dL)	118.42	126.26	117.29	119.65	1.72	0.141	0.261	0.426
High-density lipoprotein (mg/dL)	108.26	112.61	108.57	110.99	1.63	0.310	0.844	0.771
Low-density lipoprotein (mg/dL)	19.49	25.74	19.22	21.44	0.71	0.002	0.077	0.119
Calcium (mg/dL)	10.89	11.03	10.49	10.57	0.10	0.564	0.036	0.872
Phosphorus (mg/dL)	6.56	5.68	5.98	5.37	0.11	<0.001	0.015	0.452
Uric acid (mg/dL)	287.74	229.71	239.22	174.56	13.12	0.016	0.040	0.893
Aspartate aminotransferase (U/L)	46.92	64.21	54.09	65.23	2.35	0.002	0.348	0.479
Alanine aminotransferase (U/L)	0.93	0.85	0.79	0.79	0.05	0.689	0.325	0.689
Alkaline phosphatase (U/L)	4322.9 ^ab^	4478.5 ^ab^	5287.9 ^a^	3825.0 ^b^	190.71	0.078	0.670	0.030
Glutamyl transpeptidase (U/L)	19.43	21.64	18.79	20.29	0.62	0.141	0.424	0.775
Creatine kinase (U/L)	2454.5	2583.2	2017.9	2970.4	138.68	0.050	0.927	0.132

^a,b^ Means with different superscript letters differ significantly (*p* < 0.05). Ctrl, control; VitA, vitamin A; NE, necrotic enteritis.

## Data Availability

Not applicable.
